# Descending Colon Intussusception Presenting With Bleeding per Rectum

**DOI:** 10.7759/cureus.107888

**Published:** 2026-04-28

**Authors:** Mohamed Ahmed, Dorothy He, Mohammed Asaad, Mustapha Akhdar, Danya Auda

**Affiliations:** 1 Surgery, University of California, Riverside, USA; 2 Surgery, AdventHealth Tampa, Tampa, USA; 3 General Surgery, AdventHealth Tampa, Tampa, USA; 4 Psychology, University of California, Riverside, USA

**Keywords:** abdominal pain, acute gastrointestinal bleed, colonic intussusception, colon intramural lipoma, left-sided abdominal pain, submucosal lipoma

## Abstract

Intussusception happens when the proximal bowel invaginates into the distal bowel, which can compromise the blood supply and may cause bowel obstruction. Colon intussusception is rare in adults and accounts for only a small proportion of bowel obstructions. Lower gastrointestinal bleeding is rarely caused by this pathology. Intussusception may be idiopathic or the result of underlying benign or malignant pathology. The diagnosis became more reliable with the liberal use of CT. In most cases, it is recommended to respect the involved bowel segment. The patient presented here is a young, otherwise healthy female patient who presented to our hospital with abdominal pain and fresh bleeding per rectum. The CT scan revealed colon intussusception. Laparoscopic left colon resection was performed, and the pathology revealed a submucosal lipoma as the underlying cause.

## Introduction

The most commonly used definition for intussusception is the invagination of a proximal bowel segment into the lumen of an adjacent distal segment [1]. The age of presentation is highly variable, ranging from the neonatal period to the seventh decade of life [2]. The pediatric population commonly presents with abdominal pain, which is colicky in nature and not common in adults [3]. Hematochezia is an uncommon presenting symptom and is seen in less than 16% of cases with a normal physical exam in most cases [4]. The underlying cause of adult intussusception can be due to an unknown cause defined as 'idiopathic pathology' or due to benign or malignant pathology [5]. Around 5% of intussusception affects the adult population and, in 1% to 5% of cases, causes bowel obstruction; 10% to 20% of cases are idiopathic, without a lead point lesion, while secondary intussusception is caused by organic lesions, such as inflammatory bowel disease, postoperative adhesions, Meckel’s diverticulum, benign and malignant lesions, or metastatic neoplasms, or is iatrogenic, due to the presence of intestinal tubes, jejunostomy feeding tubes, or after gastric surgery [6].

## Case presentation

The patient is a 27-year-old, otherwise healthy female who presented with bleeding per rectum following a two-week history of left lower quadrant abdominal pain. Laboratory findings were within normal limits (Tables 1-2). The patient's vital signs were within normal limits, and physical exam revealed a soft, nontender abdomen.

**Table 1:** Basic metabolic panel

Parameter	Value		Reference range		
Sodium	137 mmol/l		133-143 mmol/l		
Potassium	3.7 mmol/l		3.6-5.2 mmol/l		
Chloride	103 mmol/l		98-112 mmol/l		
Carbon dioxide	26 mmol/l		21.0-32.0 mmol/l		
Anion gap	8 mmol/l		5-15 mmol/l		
Glucose	101 mg/dl		70-100 mg/dL		
Bun	6 mg/dl		7.0-18.0 mg/dL		
Creatinine	0.66 mg/dl		0.60-1.30 mg/dL		
Calcium	8.5 mg/dl		8.8-10.5 mg/dL		

**Table 2:** Complete blood count

Parameter	Value	Reference range
WBC	9.3 10*3/ul	4.80-10.80 10*3/uL
RBC	4.74 10*6/ul	4.70-6.10 10*6/uL
Hemoglobin	in 14 g/dl	14.0-18.0 g/dL
Hematocrit	40%	42.0%-54.0%
Mean corpuscular volume	84.4 fl	80.0-98.0 fL
Mean corpuscular hemoglobin	29.5 pg	27.0-31.0 pg
Mean corpuscular haemoglobin concentration	35 g/dl	32.0-36.0 g/dL
Platelet	389 10*3/ul	150-400 10*3/uL

**Figure 1:**
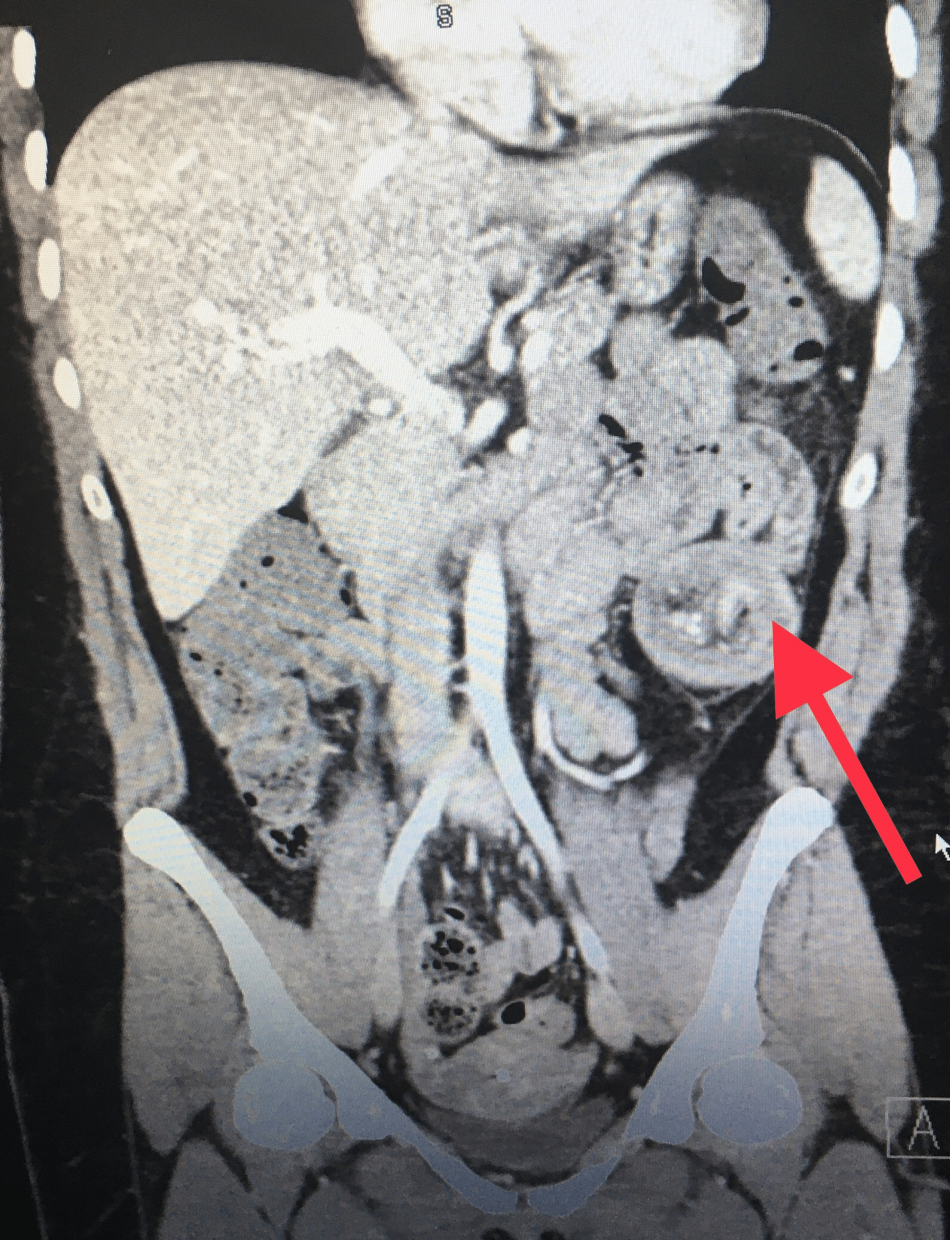
Coronal view of the CT scan of the abdomen and pelvis Red arrow: Target sign on the intussusception of the descending-sigmoid colon

**Figure 2:**
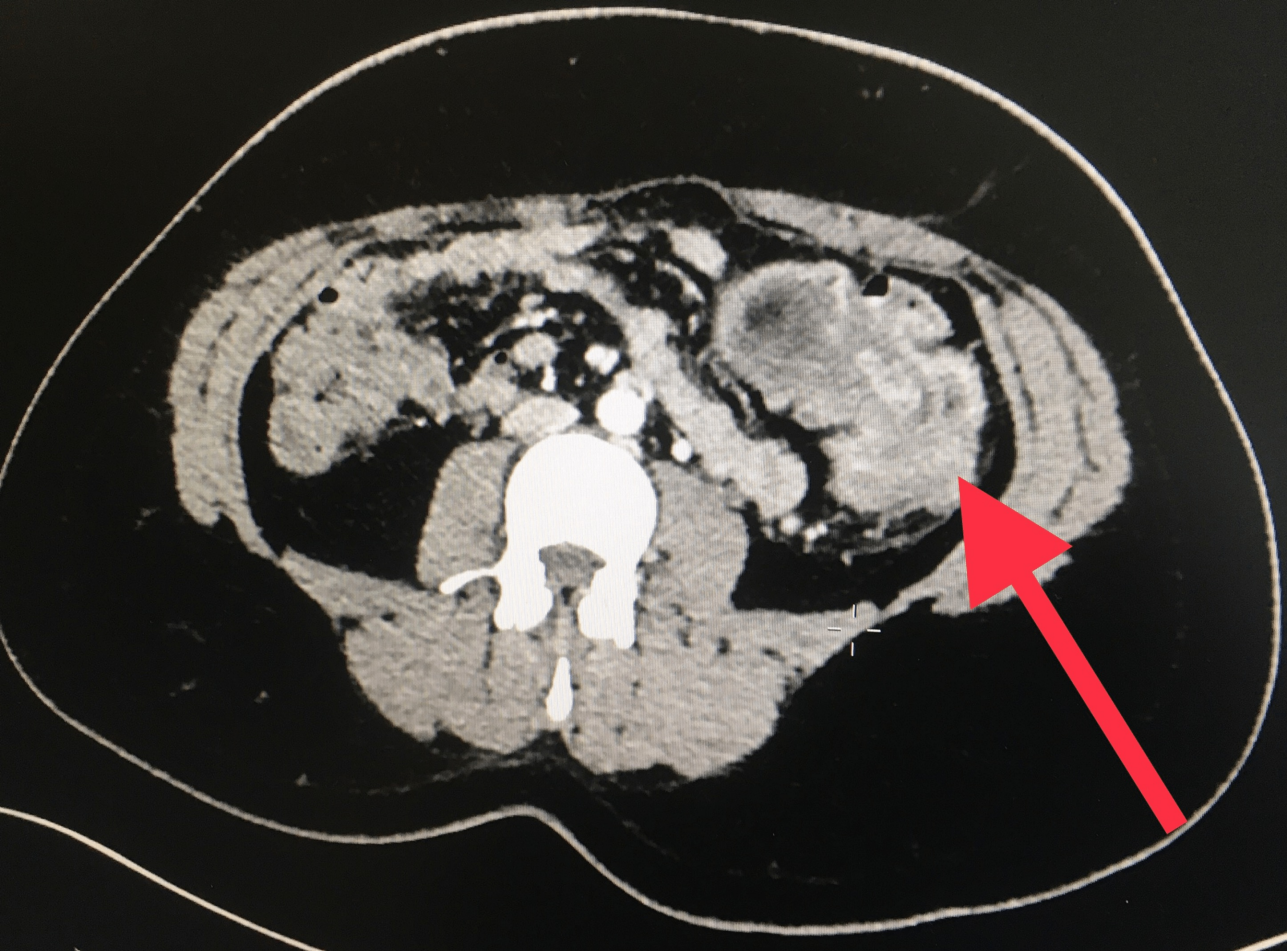
Axial view of the CT scan of the abdomen and pelvis Red arrow: Fat density on the intussusception of the descending-sigmoid colon

**Figure 3:**
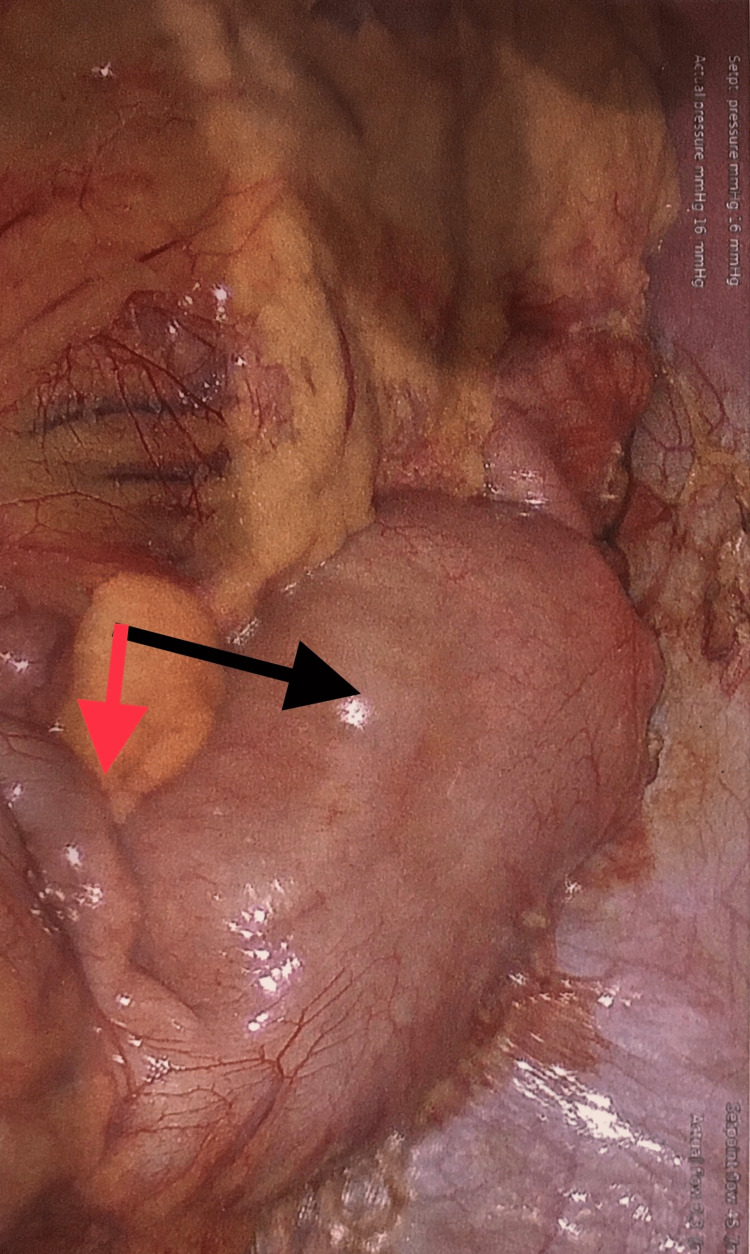
Intraoperative view Black Arrow: Descending colon intussusception; Red Arrow: Sigmoid colon

**Figure 4:**
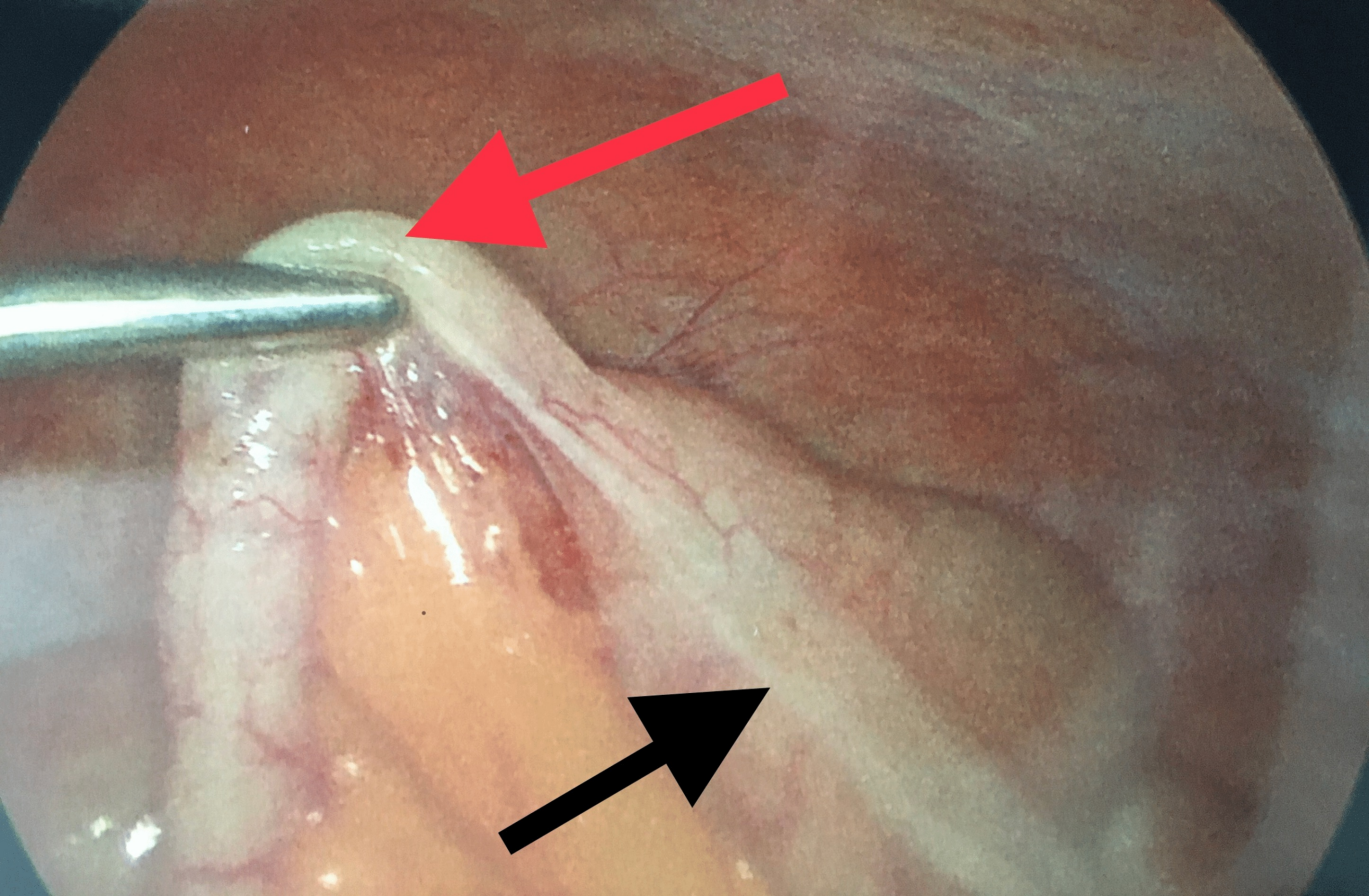
Laparoscopic view Black arrow: Descending colon intussusception; Red arrow: Sigmoid colon

A CT scan showed a 4 cm x 3 cm mass in the sigmoid colon, likely a lipoma, with resultant intussusception (Figures 1-2). Laparoscopic resection of the distal descending and proximal sigmoid colon was performed (Figures 3-4). The patient had an uneventful recovery with home discharge on postoperative day three.

## Discussion

Epidemiology and etiology

Intussusception is rare in adults, accounting for less than 5% of all cases of intussusception and almost 1% to 5% of bowel obstruction. Descending colon intussusception is reported mostly in case reports and small series and has a lower incidence due to retroperitoneal fixation, which reduces mobility [7]. Around 70% to 90% of adult patients have an organic lesion, while 90% of pediatric intussusception is idiopathic; lipomas in the intestinal tract are still relatively rare and account for only 5% of all gastrointestinal tumors. In most cases, a lipoma of the colon is localized at the submucosal level. Due to their intramural location, lipomas can also serve as the leading point for intussusceptions.

Allos et al. reported a rare case of colo-colonic intussusception in an adult secondary to descending colonic lipoma [7]. It commonly affects the small intestine in 85% of cases and the colon in 15% of cases [8]. Common causes include malignant adenocarcinoma of the colon (most common), lymphoma, metastatic lesions, benign lipoma, polyps, endometriosis, Meckel's diverticulum (more proximal), postoperative adhesions, trauma, and viral illness [9]. Idiopathic adult intussusception presenting with abdominal pain, bloody mucoid stool, and an abdominal mass has been reported as a presentation [10]. Underlying malignancy as a cause is seen in 60% to 70% of the adult population [11].

Clinical presentation

Symptoms include episodes of intermittent abdominal pain, vomiting, intermittent obstruction symptoms, constipation, rectal bleeding, or palpable abdominal mass, which is rare [12]. Bowel obstruction symptoms are the usual presentation and include abdominal pain, usually crampy in nature, in 71% of patients; nausea and vomiting in 68% of patients; abdominal fullness in 45% of patients; and abdominal tenderness in 60% of patients [13,14]. Intussusception of the rectosigmoid through the anal canal mimicking rectal prolapse secondary to a sigmoid colon submucosal lipoma has been reported [15]. Gastrointestinal bleeding in intussusception secondary to submucous lipoma has been described and may result from mucosal ischemia with erosion as a result of prolonged telescoping and vascular compromise, resulting in hematochezia or melena. Ulceration of the lead point in the case reported by Domínguez et al. was a lipoma with overlying mucosal ulceration, which resulted in gastrointestinal bleeding [16]. Since bleeding may be the presenting symptom (as in the current case), clinicians should include intussusception in the differential diagnosis for unexplained lower gastrointestinal bleeding accompanied by intermittent abdominal pain or obstructive symptoms, particularly when standard endoscopic evaluation is non-diagnostic. 

Diagnosis and management

The CT scan is the gold standard. Typical findings include the target sign, sausage-shaped mass, bowel-within-bowel appearance, mesenteric fat, and vessels inside the intussusception; CT can also identify the lead point lesion [17]. The modality of choice for diagnosing intussusception is CT imaging [18]. A colonoscopy or barium enema can diagnose the cause of intussusception and may reduce it and guide treatment decisions [19]. Surgical resection is the recommended treatment for intussusception; endoscopic resection of <2 cm of the colon lipoma is an option [20].

## Conclusions

Colonic intussusception is a rare entity in adults and typically presents with vague or nonspecific symptoms. Bowel obstruction symptoms are the usual presentation, and rarely, with lower gastrointestinal bleeding. Different test modalities can help with the diagnosis. However, CT scanning is the modality of choice, especially as adult colonic intussusception is frequently associated with underlying malignancy. Our case differs from the typical adult presentation, as it involves a younger female patient presenting with a lower gastrointestinal bleed as a result of a benign submucosal lipoma serving as the lead point. Barium enema and colonoscopy can aid in the diagnosis of intussusception and, in selected cases, may allow reduction. However, in adults, reduction is generally not recommended due to the high likelihood of an underlying malignancy. Endoscopic resection of the tumor is only recommended for benign tumors <2 cm in diameter. Almost half of colonic intussusceptions in the adult population are associated with malignancy, a reason why surgical resection without reduction is the preferred treatment.
